# Complement Receptor 1 (CR1, CD35) Polymorphisms and Soluble CR1: A Proposed Anti-inflammatory Role to Quench the Fire of “Fogo Selvagem” Pemphigus Foliaceus

**DOI:** 10.3389/fimmu.2019.02585

**Published:** 2019-11-22

**Authors:** Luana Caroline Oliveira, Gabriela Canalli Kretzschmar, Andressa Cristina Moraes dos Santos, Carolina Maciel Camargo, Renato Mitsunori Nisihara, Ticiana Della Justina Farias, Andre Franke, Michael Wittig, Enno Schmidt, Hauke Busch, Maria Luiza Petzl-Erler, Angelica Beate Winter Boldt

**Affiliations:** ^1^Laboratory of Human Molecular Genetics, Department of Genetics, Federal University of Paraná, Curitiba, Brazil; ^2^Laboratory of Molecular Immunopathology, Department of Clinical Pathology, Clinical Hospital, Federal University of Paraná, Curitiba, Brazil; ^3^Institute of Clinical Molecular Biology, Christian-Albrechts-University of Kiel, Kiel, Germany; ^4^Lübeck Institute of Experimental Dermatology, University of Lübeck, Lübeck, Germany; ^5^Department of Dermatology, University of Lübeck, Lübeck, Germany

**Keywords:** pemphigus foliaceus, complement receptor 1, CR1, CD35, polymorphism, York blood antigen, McCoy blood antigen, fogo selvagem

## Abstract

Pemphigus foliaceus is an autoimmune disease that is sporadic around the world but endemic in Brazil, where it is known as fogo selvagem (FS). Characterized by autoantibodies against the desmosomal cadherin desmoglein 1, FS causes painful erosions, and crusts that may be widespread. The recognition of antigens, including exposed sugar moieties, activates the complement system. Complement receptor 1 (CR1, CD35), which is responsible for the Knops blood group on erythrocytes (York and McCoy antigens), is also expressed by antigen-presenting cells. This regulates the complement system by removing opsonized antigens, blocking the final steps of the complement cascade. Membrane-bound CR1 also fosters antigen presentation to B cells, whereas soluble CR1 has anti-inflammatory properties. *CR1* gene polymorphisms have been associated with susceptibility to complex diseases. In order to investigate the association of *CR1* polymorphisms with FS susceptibility, we developed a multiplex sequence-specific assay to haplotype eleven polymorphisms in up to 367 FS patients and 242 controls from an endemic area and 289 from a non-endemic area. We also measured soluble CR1 (sCR1) in the serum of 53 FS patients and 27 controls and mRNA levels in the peripheral blood mononuclear cells of 63 genotyped controls. The haplotypes CR1^*^3B2B (with the York antigen–encoded by p.1408Met) and CR1^*^3A2A (with p.1208Arg) were associated with protection against FS (OR = 0.57, *P* = 0.027, and OR = 0.46, *P* = 0.014, respectively). In contrast, the CR1^*^1 haplotype (with the McCoy antigen – encoded by p.1590Glu) was associated with FS susceptibility (OR = 4.97, *P* < 0.001). Heterozygote rs12034383^*^A/G individuals presented higher mRNA expression than homozygotes with the *G* allele (*P* = 0.04). The lowest sCR1 levels occurred in patients with active disease before treatment (*P* = 0.036). Patients in remission had higher levels of sCR1 than did healthy controls (*P* = 0.013). Among those under treatment, patients with localized lesions also presented higher sCR1 levels than those with generalized lesions (*P* = 0.0073). In conclusion, the Knops blood group seems to modulate susceptibility to the disease. Furthermore, corticosteroid treatment might increase sCR1 serum levels, and higher levels may play an anti-inflammatory role in patients with FS, limiting the distribution of lesions. Based on these results, we suggest CR1 as a potential new therapeutic target for the treatment of FS.

## Introduction

Pemphigus foliaceus (PF) is an autoimmune blistering skin disease presenting with erosions and crusts that may be widespread and painful (1). Around the world, PF occurs sporadically, with an incidence of 0.75–5 cases/million per year (2, 3). In Brazil, it reaches up to 3% prevalence and is also known as *fogo selvagem* (FS, meaning “wild fire” in Portuguese) (2–6). While major immunopathological and histological characteristics are similar in both endemic and sporadic forms, the clinical presentation may differ (3, 7). The etiology of FS is little understood, but environmental factors are being considered. The bites of black mosquitoes (*Simuliidae*) and sand flies (*Phlebotominae*) are associated with an almost five times increased susceptibility to FS. Elements delivered in the saliva of these hematophagous insects are thought to trigger a cross-reaction against keratinocyte surface epitopes in genetically susceptible individuals living in endemic regions (8–10).

The intraepidermal blisters of PF are due to keratinocyte detachment, a process known as acantholysis (11). FS patients generate pathogenic IgG4 autoantibodies against desmoglein 1 (Dsg1) and occasionally and to a much lesser extent, against Dsg3, which are important structural components of desmosomes (1, 12). Altered cell-associated molecular patterns activate the complement system, and this is responsible for the recurrent observation of C3 component deposits along the basement membrane zone and in intercellular spaces of the perilesional and lesional epidermis of FS patients (13–16). Deposits of complement membrane attack complexes, in contrast, were only found in injured skin, whereas pathogenic IgG4 anti-desmoglein 1 autoantibodies—which cannot activate complement—were also abundant in apparently healthy tissue in FS patients (14, 17).

Although not required to initiate blister formation (18, 19), complement activation enhances acantholysis in cell culture (20, 21). Furthermore, FS patients with active disease present enhanced expression of the *C1QA* gene (an initiator molecule of the classical pathway) and increased serum levels of C3 and C-reactive protein (opsonins), of the cleaved factors resulting from the activation of the alternative pathway (Ba factor), or of the classical/lectin complement pathways (C4d factor), as well as a trend to decreased mannose-binding lectin serine protease 2 levels, reflecting activation of the lectin pathway (14, 22–24).

**Figure 1:**
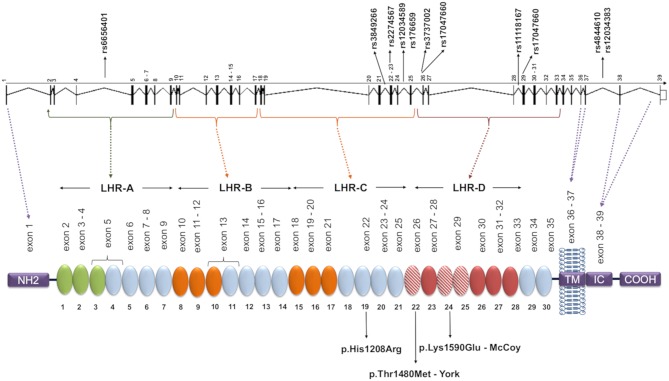
*CR1* gene and protein structure and localization of the investigated SNPs. Each circular block represents a SCR (short consensus repeat, numbered 1–30), encoded by the exons 2–35, listed immediately above. There are three C4b binding sites (SCR 1-3, 8-10, and 15-17, in green and orange) and two C3b binding sites (SCR 8-10 and 15-17, both in orange). SCRs 22-28 bind C1q, MBL, and ficolins, and SCRs 22, 24, and 25 (red dashed blocks) carry Knops blood group antigens. The SNPs analyzed in this study are indicated in the gene, and the amino acid substitutions resulting from polymorphisms located in exons are indicated on the protein. The dashed lines indicate coding exons of the main parts: the aminoterminal region (NH2, exon 1), transmembrane domain (TM, exons 36-37), the intracytoplasmic carboxi-terminal domain (IC COOH, exons 38-39), and the four long homologous repeats (LHR), responsible for complement decay-accelerating, and cofactor activities (exons 1-7, 8-14, 15-21, 22-28). The functional sites in 8-10 and 15-17 are nearly identical. Repeats in green are required for C4b binding and decay-accelerating activity, while those in orange are required for C3b and C4b binding and cofactor activity [adapted of (25)].

Complement receptor 1 (CR1, CD35) is a glycoprotein with ~200 kDa encoded by the *CR1* gene (on chromosome region 1q32). The extracellular domain of the most common form of CR1 is composed of a series of 30 repeating units named short consensus repeats (SCRs). The SCRs are distributed in four long homologous repeats (LHRs A, B, C, and D), arising from duplication of a seven-SCR unit (Figure 1) (26–28). CR1 is primarily expressed in erythrocytes, B and T cells, neutrophils, monocytes, and dendritic cells, as well as in neurons, microglia, and the choroid plexus of the brain, in the membrane or in soluble form (29). The soluble CR1 (sCR1) form results from proteolytic cleavage in terminal secretory vesicles or in the cell membrane (30, 31). Both CR1 forms act to regulate complement activity by binding cleaved C3b and C4b components as well as the complement cascade initiation molecules mannose-binding lectin (MBL-2), ficolins (FCN1, FCN2, and FCN3), and C1q deposited in altered cell components or pathogens. CR1 competes with serine proteases (MASPs) for the same binding sites on the collagenous tails of MBL and FCNs, inhibiting the initiation of the lectin pathway of complement (32). Upon binding, the membrane-bound form of CR1 internalizes the opsonized elements or presents them to other immune cells, preventing the formation of the C5 convertase. This blocks the formation of the membrane attack complex (MAC). CR1 might also prevent excessive complement activation, acting as a cofactor for the Factor I-mediated cleavage of soluble/bound C3b and C4b (33). On the other hand, binding of opsonized elements fosters antigen presentation to lymphocytes, increasing antibody production, and the humoral response (29, 34). In the presence of excess interleukin 2, CR1 expressed on activated T lymphocytes generates regulatory T cells in secondary lymphoid organs, where they may interact with B cells (35). In fact, CR1 ligation inhibits B cell receptor-mediated activation and differentiation to plasma cells (36).

In a recent study by our group that encompassed polymorphisms of all genes encoding complement components, we found evidence for an association with gene variants of almost all complement elements previously detected in the epidermis or with altered serum levels in FS patients. In addition, we identified the association of four opsonin-binding complement receptors (CR1-4, encoded by *CR1, CR2, ITGAM*, and *ITGAX*) (37). Among them, we found that a *CR1* haplotype with the major rs6656401^*^G allele was associated with increased susceptibility to FS (37). This prompted us to thoroughly investigate the *CR1* gene for other polymorphisms, including those encoding blood group antigens that may affect the binding of C1q, MBL, and FCNs, as well as to measure the expression of this gene at the mRNA and soluble protein levels.

## Materials and Methods

### Ethics Statement

This transversal case-control study was approved by the National Committee for Ethics in Research (CONEP 02727412.4.0000.0096, protocol 505.988). All study participants were informed about the research and signed a term of informed consent.

### Research Participants

For the association study, a total of 367 FS patients and 242 controls from an endemic area and 289 individuals from a non-endemic area (among accompanying persons, health workers, and other volunteers) were analyzed. The participants were recruited from four centers: Hospital Adventista do Pênfigo (Campo Grande-MS, Central Western Brazil), Lar da Caridade (Uberaba-MG, Southwestern Brazil), Hospital das Clínicas da Faculdade de Medicina de Ribeirão Preto (Ribeirão Preto-SP, Southwestern Brazil), and Hospital das Clínicas da Universidade Federal do Paraná (UFPR) (Curitiba-PR, South Brazil). Data for both patients and controls were collected through interviews and medical records. Inclusion criteria were: written informed consent, being a resident in the endemic region, and for the patients, FS diagnosis based on clinical evidence and positive anti-Dsg1 serology. Exclusion criteria were: consanguinity with a control or a patient and a familial history of autoimmune diseases. All subjects were classified according to ancestral origin, based on physical characteristics and self-reported ancestry, as previously described (38, 39). The demographic characteristics of the participants in this study are listed in Table 1.

**Table 1:** Demographic data on endemic pemphigus foliaceus patients and controls.

	**Controls**		**FS patients**		
	**Endemic *n* = 204**	**Non-endemic *n* = 256**	**Patients *n* = 265**	**Localized lesions *n* = 44**	**Generalized lesions *n* = 80**
**Epidemiologic data**					
Male (%)	99 (48.5)	106 (41.4)	121 (45.7)	18 (40.9)	32 (40.0)
Average age (min-max)	44.75 (13–86)	33.64 (17–66)	37.42 (12–88)	28.5 (16–73)	38.55 (14–85)
**Demographic data**					
Central-Western (%)	177 (86.8)	0	206 (77.7)	31 (70.5)	57 (71.3)
Southwestern (%)	5 (2.5)	0	56 (21.1)	12 (27.3)	21 (26.3)
South (%)	22 (10.8)	256 (100)	3 (1.1)	1 (2.3)	2 (2.5)

*N, number of individuals; Central-Western, Campo Grande-MS; Southwestern, Ribeirão Preto-SP. South, Curitiba-PR*.

*The total number of patients with localized or generalized lesions did not sum 265 due to lack of proper diagnostic classification of some individuals*.

For the analysis of sCR1 levels in serum, we selected 53 FS patients and 27 healthy controls, matched for sex, age, and previously genotyped for *CR1* polymorphisms. Eighteen of the patients were in complete remission, nine were without treatment and nine were under immunosuppressive treatment; 35 patients presented with active fogo selvagem, six of whom were still untreated and 29 of whom were already being treated. Among all treated patients with known information regarding the distribution of lesions, five had localized lesions, and 21 had generalized lesions.

In order to evaluate a possible correlation between *CR1* polymorphisms and mRNA levels from peripheral blood mononuclear cells (PBMC), we also genotyped 158 healthy unrelated Euro-Brazilian volunteers, resident in Curitiba, Brazil, and surrounding areas. We selected 63 individuals from this group with the following inclusion criteria: no previous history of an autoimmune disease, an obvious skin disease, and presenting *CR1* alleles and haplotypes associated with the disease (to quantify mRNA levels).

### *CR1* Genotyping

Blood was collected with anticoagulant EDTA, and DNA was extracted from PBMC as previously described, using phenol-chloroform-isoamyl alcohol (40).

We selected 11 single nucleotide polymorphisms (SNPs) based on [1] being previously associated with a disease, [2] being a tag SNP with r^2^ ≥ 0.8 in the European (Utah—USA), Mexican, Colombian, or Yoruba populations of the 1000 Genomes Project (41); and/or [3] by presenting a minor allele frequency higher than 0.05. The *CR1* SNPs evaluated were: rs6656401^*^a>*G* (intron 4); rs3849266^*^c>*T* (intron 21); rs2274567^*^a>*G* (exon 22, p.His1208Arg); rs12034598^*^a>*G* (intron 24), rs1746659^*^t>*A* (intron 24), rs3737002^*^c>*T* (exon 26, p.Thr1408Met, responsible for the York blood group antigen); rs11118131^*^c>*T* (intron 26); rs11118167^*^t>*C* (intron 28); rs17047660^*^a>*G* (exon 29, p.Lys1590Glu, responsible for the McCoy blood group antigen); rs4844610^*^a>*C* (intron 37); rs12034383^*^g>*A* (intron 37).

We developed two multiplex Polymerase Chain Reaction-Sequence Specific Primer (PCR-SSP) reactions for simultaneous identification of four SNPs and two simple PCR-SSPs for one SNP each. All PCRs co-amplified a Human Growth Hormone (*HGH*) or Human Leukocyte E antigen (*HLA-E*) fragment as internal control. The sequences of specific and control primers are shown in Table 2. All reactions were carried out in a final volume of 8 μl, containing 20 ng of genomic DNA, 0.2 mM each of dNTP and 1x Coral Buffer (Invitrogen Life Technologies, Carlsbad, USA) (Supplementary Table 1). Thermal cycling began with 95°C for 5 min, followed by 33 (PCR-SSP-1) or 30 (PCR-SSP-2, 3 and 4) cycles, for which each cycle began with 94°C for 20 s and ended with 72°C (extension step) for 40 s (Supplementary Table 1). We submitted the amplified fragments to an electrophoretic run on 1% agarose gel, stained with Sybersafe (Invitrogen Life Technologies, Carlsbad, USA).

**Table 2:** Primers for *CR1* sequence-specific amplification.

	**Forward Primers**			**Reverse primers**			
	**SNP**	**Localization**	**5^**′**^ → 3^**′**^ Sequence**	**SNP**	**Localization**	**5^**′**^ → 3^**′**^ Sequence**	**Fragment**
**Simple PCR 1**	**rs6656401**	Intron 4	CTCTGTCTCCATCTTCTC**A**	**Generic Primer**	Intron 4	CATAGTTGTAGTTGGGGATT**G**	257 bp
			CTCTGTCTCCATCTTCTC**G**				
**Multi 1**	**rs3849266**	Intron 21	CTGATGGCTTGGGGTA**T**	**rs2274567**	Exon 22	CTCAATCTGCATTGATCCA**C**	667 bp
			CTGATGGCTTGGGGTA**C**			CTCAATCTGCATTGATCCA**T**	
**Simple PCR 1**	**rs12034598**	Intron 24	GCCTGGCATCATCAAGAAA**A**	**rs1746659**	Intron 24	GGTTAGTGTTCAGAGGCAG**A**	1068 bp
			GCCTGGCATCATCAAGAAA**G**			GGTTAGTGTTCAGAGGCAG**T**	
**Multi 2**	**rs3737002**	Exon 26	CCATTTGCCAGTCCTA**C**	**rs11118131**	Intron 26	CAAGAAGAAGGGGTGAT**G**	457 bp
			CCATTTGCCAGTCCTA**T**			CAAGAAGAAGGGGTGAT**A**	
**Multi 2**	**rs11118167**	Intron 28	GCCAATATGTGAATATTATTATCTTA**T**	**rs17047660**	Exon 29	TTCTGGAGCTGTGCATT**T**	746 bp
			GCCAATATGTGAATATTATTATCTTA**C**			TTCTGGAGCTGTGCATT**C**	
**Multi 1**	**rs4844610**	Intron 37	CTACACAAAACAGCCTTGT**A**	**rs12034383**	Intron 37	AGATGTCCATGCCTTAA**C**	1080 bp
			CTACACAAAACAGCCTTGT**C**			AGATGTCCATGCCTTAA**T**	
	**Control primers**						
**Simple PCR 1, 2 and muli 1**		*HGH*f	TGCCTTCCCAACCATTCCCTTA		*HGH*r	CCACTCACGGATTTCTGTTGTGTTTC	431 bp
**Multi 2**		*HLA-E*f	CGGGACTGACTAAGGGGCGG		*HLA-E*r	GTAGCCCTGTGGACCCTCTTAC	324 bp

*In bold: variant nucleotide. SNP, single nucleotide polymorphism; bp, base pairs; HGH, human growth hormone; HLA-E, Human leukocyte E antigen; f, forward; r, reverse; Multi, multiplex PCR-SSP*.

Genotyping of the rs6656401, rs3849266, rs3737002, rs11118131, rs11118167, rs17047660, and rs12034383 polymorphisms was performed with the iPLEX platform of the MassARRAY system (Agena Bioscience, San Diego, USA) at the Insitut für Klinische Molekularbiologie (IKMB), Christian-Albrechts-Universität, Kiel, Germany (42), in order to technically validate the results obtained by PCR-SSP. Genotypes were called using the software MassARRAY Typer (v4.0) (Agena Bioscience, San Diego, USA) with standard settings. The sequences of specific and control primers are shown in Table 3.

**Table 3:** Primers for the *CR1* Sequenom massARRAY iPLEX Platform.

**SNP**	**Localization**	**5^**′**^ → 3^**′**^ Forward Primer Sequence**	**5^**′**^ → 3^**′**^ Reverse Primer Sequence**	**5^**′**^ → 3^**′**^ Extended Primer Sequence**
rs6656401	Intron 4	ACGTTGGATGGCATCATTTCCTTCTCTGTC	ACGTTGGATGGACAGAAGAGCAAAGGACAC	GTTTTTTCTCTGTCTCCATCTTCTC
rs3849266	Intron 21	ACGTTGGATGATAACTCGCCCCTCAATCTG	ACGTTGGATGGCAGGCAACTGTGTTTATAC	ACCTCAATCTGCATTGATCCA
rs3737002	Exon 26	ACGTTGGATGTCCAGGTCACTGTAAAACCC	ACGTTGGATGAAGATGTCCCGACTGGAAAC	CTATCAGTTTCCATTTGCCAGTCCTA
rs11118131	Intron 26	ACGTTGGATGGATGTCTATATTTGCCCAAG	ACGTTGGATGGCATCTAGTCAAGGTTCAGG	GGTATAAAGGAAAGGATGGTCTTGTA
rs11118167	Intron 28	ACGTTGGATGATGTGTGTAGTCACTTAGCC	ACGTTGGATGATAATGGCAGATTTAAGGGC	GCCAATATGTGAATATTATTATCTTA
rs17047660	Exon 29	ACGTTGGATGCAAGTTGGTGTTTGGAGCAG	ACGTTGGATGGCATTTTCAACTTCTGGAGC	TCACCTTCTGGAGCTGTGCATT

*SNP, single nucleotide polymorphism*.

### sCR1 Quantification

We measured sCR1 levels in the serum (collected without anticoagulant) by using a SEB123Hu ELISA kit (USCN Life Science Inc., Wuhan, China) according to the manufacturer's instructions. Anti-Dsg1 and anti-Dsg3 levels were previously measured using commercial ELISA kits (RG-M7593-D and RG-7680-EC-D, respectively) (MBL, Woburn, USA) (43).

### mRNA Quantification

Total RNA was isolated from PBMC lysed in TRIzol (Ambion, Austin, USA) and reverse-transcribed with a High Capacity cDNA Reverse Transcription Kit (Applied Biosystems, Foster City, USA). Gene expression levels were quantified by qPCR using TaqMan (Applied Biosystems, Foster City, USA) chemistry for the *CR1* (Hs00559348_m1, the most common CR1 transcript) and the housekeeping Glucuronidase Beta (*GUSB)* gene (4333767F). All assays were performed in triplicate, and the relative mRNA levels were normalized for mRNA expression of the *GUSB* gene. Cq values (threshold cycle) were calculated using the Viia 7 Software v1.2 Kit (Applied Biosystems, Foster City, USA), and gene expression was calculated via the comparative Cq method 2-ΔΔCq (44).

### Statistical and Bioinformatics Analysis

We obtained allele, genotype and two-SNP haplotype frequencies by direct counting, and the hypothesis of Hardy-Weinberg equilibrium was calculated with the exact test of Guo & Thompson. We also compared haplotype distribution between the investigated groups, using the exact test of population differentiation of Raymond and Rousset. All tests were performed in Arlequin v.5.1, a population genetics software package (45).

**Figure 2:**
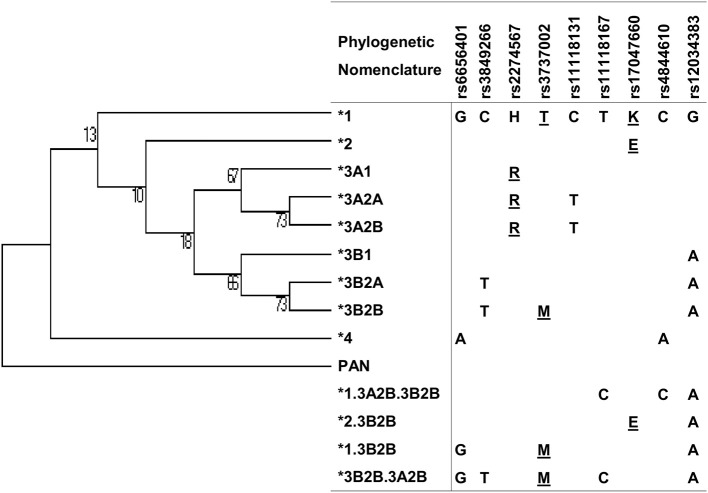
Maximum parsimony tree of *CR1* haplotypes with nucleotide changes and phylogenetic nomenclature. Underlined: amino acid substitutions.

We reconstructed extended haplotypes based on linkage disequilibrium (LD) and phase information about the two-SNP haplotypes obtained by PCR-SSP amplification, compared the results with the haplotypes estimated using the EM algorithm (Expectation-Maximization) implemented in PLINK software (46), and found 2.9% of haplotypes with discrepant results in accordance with the literature (47). We also used PLINK for analysis of possible SNP interactions and evaluated LD with Haploview 4.2 (48). We used Mega Software v.6 to name the haplotypes according to the phylogenetic nomenclature suggested by the literature (49) and adapted them for recombinant haplotypes (Figure 2). We further compared the distribution of polymorphisms and haplotypes in patients and controls adjusted for the proportion of ancestry groups using the Fisher exact test in the online software VassarStats (VassarStats: Website for Statistical Computation; available at: http://vassarstats.net). We adjusted all associations for the possible effects of confounding factors (age, sex, ancestry group) by logistic regression in total patient and control groups using the software STATA v.9.2 (Statacorps, Lakeway Drive, USA). To check for false discovery rate, we used the correction of Benjamini and Hochberg (50) on all significant results (“q” values lower than 0.05 were considered significant).

Normality tests (D'Agostino & Pearson and Shapiro-Wilk), correlation tests (Spearman), and non-parametric comparisons of *CR1* mRNA and sCR1 levels (Mann-Whitney and Kruskal-Wallis tests) were carried out using GraphPad Prism v.5.01 (GraphPad Software). The use of different dosages of corticosteroids was evaluated in the group of patients undergoing treatment in order to verify whether there were differences between them.

To predict the functional effects of associated SNPs, we explored data from the ENCODE project contained in the Regulome-DB (51) and Haploreg (52) databases. To evaluate the effect of non-synonymous SNPs, we used Polyphen-2 (53) and SNiPA (54).

## Results

### *CR1* Polymorphisms and Susceptibility to Fogo Selvagem

None of the groups had genotype distributions that deviated from those predicted under Hardy-Weinberg equilibrium. The allele frequencies for each SNP did not differ between controls and the Iberian population from the 1,000 Genomes Project. In contrast, *CR1* haplotype distributions differed between the endemic and non-endemic controls, even within each ancestry group (*P* < 0.0001). This led us to compare the patient group with endemic and non-endemic control groups separately. We found differences between the allelic distributions of isolated SNPs of non-endemic controls and patients.

Comparing these two groups (Table 4, Supplementary Table 2), we found evidence for a disease association with the minor allele of rs17047660^*^G in exon 29. This variant encodes p.1590Glu, which is responsible for positive McCoy blood group antigen. Its allelic frequency was higher among FS patients [OR = 3.77 (95%CI = 1.52–9.35), *q* = 0.005]. rs17047660^*^A/G heterozygote individuals were more frequent among those with generalized lesions [OR = 3.06 (95%CI = 1.09–8.56), *q* = 0.025]. The rs3849266 within intron 21 occurs in a region with enhancer histone H3K4me1 marks, binding at least 23 different regulatory proteins, according to the ENCODE Database. rs3849266^*^C/T presented an association with increased susceptibility to the disease [OR = 1.54 (95%CI = 1.03–2.31), *q* = 0.030]. We also found an additive effect of carrying the “wild type” haplotype with rs3737002^*^C in exon 26 (p.1408Thr, York negative) and rs11118131^*^C in intron 26 [OR = 1.5 (95%CI = 1.00–2.26), *q* = 0.049] with susceptibility to severe FS. The minor allele rs2274567^*^G, encoding p.1208Arg, occurred more frequently among non-endemic controls than among patients [OR = 0.58 (95%CI = 0.38–0.87), *q* = 0.035].

**Table 4:** Association of *CR1* variants with endemic pemphigus foliaceus.

		**Controls**		**FS patients**				
***CR1* genetic variants**	**Iberian *n* = 214 (%)**	**Endemic control *n* (%)**	**Non-endemic control *n* (%)**	**Patient *n* (%)**	**Localized lesion *n* (%)**	**Generalized lesion *n* (%)**	**Patient vs. Non-endemic control**	
***rs6656401 A>G***		*n* = 203	*n* = 234	*n* = 265	*N* = 44	*N* = 81	**OR [95% CI]**	**P (q) value**
*A*	27 (12.60)	46 (11.33)	63 (13.46)	59 (11.13)	8 (9.09)	20 (12.35)		
*AA*	2 (1.90)	2 (0.99)	1 (0.43)	2 (0.75)	1 (2.27)	0		
*AG*	23 (21.50)	42 (20.69)	61 (26.07)	55 (20.75)	6 (13.64)	20 (24.69)		
*GG*	82 (76.60)	159 (78.33)	172 (73.50)	208 (78.49)	37 (84.09)	61 (75.31)		
*A+*		44 (21.67)	62 (26.50)	57 (21.51)	7 (15.91)	20 (24.69)		
***rs3849266 C>T***		*n* = 182	*n* = 238	*n* = 233	*n* = 41	*n* = 76		
*T*	68 (31.80)	103 (28.30)	122 (25.63)	125 (26.82)	19 (23.17)	38 (25.00)		
*CC*	50 (46.70)	90 (49.45)	134 (56.30)	120 (51.50)	24 (58.54)	41 (53.95)		
*CT*	46 (43.00)	81 (44.51)	86 (36.13)	101 (43.35)	15 (36.59)	32 (42.11)	1.54 [1.03–2.31]	0.036 (0.030)
*TT*	11 (10.30)	11 (6.04)	18 (7.56)	12 (5.15)	2 (4.88)	3 (3.95)		
*T+*		92 (50.55)	104 (43.70)	113 (48.50)	17 (41.46)	35 (46.05)		
***rs2274567 A>G*** **(p.His1208Arg)**		*n* = 148	*n* = 232	*n* = 158	*n* = 28	*n* = 54		
*G*	36 (16.80)	65 (21.96)	138 (29.74)	73 (23.10)	15 (26.79)	22 (20.37)	0.70 [0.51–0.98]	0.048 (0.035)
*AA*	73 (68.20)	94 (63.51)	110 (47.41)	93 (58.86)	14 (50.00)	32 (59.26)		
*AG*	32 (29.90)	43 (29.05)	106 (45.69)	57 (36.08)	13 (46.43)	22 (40.74)		
*GG*	2 (1.90)	11 (7.43)	16 (6.90)	8 (5.06)	1 (3.57)	0		
*G+*		54 (36.49)	122 (52.59)	65 (41.14)	14 (50.00)	22 (40.74)		
***rs3737002 C>T*** **(p.Thr1408Met)**		n = 192	n = 246	n = 253	n = 43	n = 79		
*T*	68 (31.80)	109 (28.39)	134 (27.24)	127 (25.10)	18 (20.93)	36 (22.78)		
*CC*	50 (46.70)	103 (53.65)	133 (54.07)	144 (56.92)	27 (62.79)	47 (59.49)		
*CT*	46 (43.00)	69 (35.94)	92 (37.40)	91 (35.97)	14 (32.56)	28 (35.44)		
*TT*	11 (10.30)	20 (10.42)	21 (8.54)	18 (7.11)	2 (4.65)	4 (5.06)		
*T+*		89 (46.35)	113 (45.93)	109 (43.08)	16 (37.21)	32 (40.51)		
***rs11118131 C>T***		*n* = 177	*n* = 208	*n* = 225	*n* = 49	*n* = 71		
*T*	35 (16.40)	75 (21.19)	103 (24.76)	89 (19.78)	15 (19.23)	26 (18.31)	0.74 [0.54–1.03]	0.08
*CC*	74 (69.20)	114 (64.41)	120 (57.69)	147 (65.33)	25 (64.10)	45 (63.38)		
*CT*	31 (29.00)	51 (28.81)	73 (35.10)	67 (29.78)	13 (33.33)	26 (36.62)		
*TT*	2 (1.90)	12 (6.78)	15 (7.21)	11 (4.89)	1 (2.56)	0		
*T+*		63 (35.59)	88 (42.31)	78 (34.67)	14 (35.90)	26 (36.62)		
***rs11118167 T>C***		*n* = 179	*n* = 232	*n* = 234	*n* = 41	*n* = 77		
*C*	35 (16.40)	65 (18.16)	91 (19.61)	82 (17.52)	18 (21.95)	28 (18.18)		
*CC*	2 (1.90)	4 (2.23)	5 (2.16)	7 (2.99)	2 (4.88)	2 (2.60)		
*CT*	31 (29.00)	57 (31.84)	81 (34.91)	68 (29.06)	14 (34.15)	24 (31.17)		
*TT*	74 (69.20)	118 (65.92)	146 (62.93)	159 (67.95)	25 (60.98)	51 (66.23)		
*C+*		61 (34.08)	86 (37.07)	75 (32.05)	16 (39.02)	26 (33.77)		
***rs17047660 A>G*** **(p.Lys1590Glu)**		*n* = 185	*n* = 235	*n* = 247	*n* = 42	*n* = 77		
*G*	3 (1.40)	10 (2.70)	6 (1.28)	23 (4.66)	4 (4.76)	9 (5.84)	3.77 [1.52–9.35]	0.002 (0.005)
*AA*	104 (97.20)	175 (94.59)	229 (97.45)	224 (90.69)	38 (90.48)	68 (88.31)		
*AG*	3 (2.80)	10 (5.41)	6 (2.55)	23 (9.31)	4 (9.52)	9 (11.69)	3.56 [1.37–9.27]	0.009 (0.015)
*GG*	0	0	0	0	0	0	2.38 [1.01–5.62]	0.048 (0.035)
*G+*		10 (5.41)	6 (2.55)	23 (9.31)	4 (9.52)	9 (11.69)	2.93 [1.18–7.24]	0.020 (0.020)
***rs4844610 A>C***		*n* = 203	*n* = 234	*n* = 265	*n* = 44	*n* = 81		
*A*	23 (10.70)	47 (11.58)	63 (13.46)	59 (11.13)	8 (9.09)	20 (12.35)		
*AA*	1 (0.90)	2 (0.99)	1 (0.43)	2 (0.75)	1 (2.27)	0		
*AC*	21 (19.60)	43 (21.18)	61 (26.07)	55 (20.75)	6 (13.64)	20 (24.69)		
*CC*	85 (79.40)	158 (77.83)	172 (73.50)	208 (78.49)	37 (84.09)	61 (75.31)		
*A+*		45 (22.17)	62 (26.50)	57 (21.51)	7 (15.91)	20 (24.69)		
***rs12034383 G>A***		*n* = 191	*n* = 242	*n* = 252	*n* = 43	*n* = 78		
*G*	71 (33.20)	188 (49.21)	231 (47.73)	249 (49.40)	46 (53.49)	82 (52.56)	3.95 [1.08–14.34]	0.029
*AA*	44 (41.10)	50 (26.18)	70 (28.93)	69 (27.38)	10 (23.26)	17 (21.79)		
*AG*	55 (51.40)	94 (49.21)	113 (46.69)	117 (46.43)	20 (46.51)	40 (51.28)		
*GG*	8 (7.50)	47 (24.61)	59 (24.38)	66 (26.19)	13 (30.23)	21 (26.92)		
*G+*		141 (73.82)	172 (71.07)	183 (72.62)	33 (76.74)	61 (78.21)		

*All genotype distributions were in Hardy–Weinberg equilibrium. Associations were corrected for age, sex, and, if independent, for ancestry group distribution. P values were checked for false discovery rate using the correction of Benjamini and Hochberg (“q” statistical significance level remained 0.05). n, number of individuals; OR, odds ratio in binary logistic regression; CI, confidence interval; q, Benjamini-Hochberg corrected p-value*.

There was strong pairwise LD between the SNPs in intron 21 (rs3849266), exon 22 (rs2274567), exon 26 (rs3737002), and intron 37 (rs12034383) (Supplementary Figure 1). Based on LD and phase information, we haplotyped all SNPs in a subset of 146 FS patients and 126 controls from an endemic area and 156 from a non-endemic area and identified 21 haplotypes (Figure 2). In fact, we obtained the most conspicuous association results with *CR1* haplotypes (Table 5), revealing synergistic, additive/epistatic effects between variants located on the same DNA string. The carrier status of the ^*^3B2B haplotype (*GTHMCTKCA*), the most common haplotype encoding the York blood group antigen (rs3737002, p.1408Met), protects against FS [OR = 0.57 (95%CI = 0.35–0.93), *q* = 0.042] as well as against the localized form of the disease [OR = 0.25 (95%CI = 0.089–0.72), *q* = 0.01]. In contrast with all other results, this association agreed for comparisons with both endemic and non-endemic controls. A similar protective effect was observed from carrying the ^*^3A2A (*GCRTTTKCG*) haplotype, presenting p.1208Arg and p.1408Thr [OR = 0.46 (95%CI = 0.25–0.85), *q* = 0.033]. This protective effect agreed with the aforementioned association results of rs2274567 (p.His1208Arg). In contrast, the frequency of the ^*^1 haplotype (*GCHTCTKCG*) was higher among FS patients with both localized and generalized distributions of cutaneous lesions than in controls (OR = 5.50 [95%CI = 1.84–16.45], *q* = 0.016 and OR = 4.33 [95%CI = 1.74–10.77], *q* = 0.002, respectively).

**Table 5:** Association of *CR1* haplotypes with endemic pemphigus foliaceus.

**Phylogenetic Haplotype**	**Nucleotide sequence**	**Patient groups n/n total (%)**	**Control groups n/n total (%)**	**OR**	**95% CI**	**P (q)**	**Model**
^*^3B2B	*GTHMCTKCA*	Patient 57/292 (19.5)	Endemic 66/252 (26.2)	0.57	0.35–0.93	0.027 (0.042)	Dominant
		Localized lesion 5/25 (20)	Endemic 66/252 (26.2)	0.25	0.089–0.72	0.010 (0.025)	Dominant
		Localized lesion 5/25 (20)	Endemic 66/252 (26.2)	0.26	0.093–0.72	0.010 (0.029)	Additive
		Localized lesion 5/25 (20)	Non-endemic 71/312 (22.8)	0.35	0.12–1.00	0.052 (0.050)	Dominant
		Localized lesion 5/25 (20)	Non-endemic 71/312 (22.8)	0.35	0.12–0.99	0.049 (0.045)	Additive
^*^1	*GCHTCTKCG*	Patient 36/292 (12.3)	Non-endemic 32/252 (12.7)	4.97	2.13–11.59	<103 (0.004)	Dominant
		Patient 36/292 (12.3)	Non-endemic 32/252 (12.7)	4.76	2.08–10.87	<103 (0.008)	Additive
		Generalized lesion 9/52 (17.3)	Non-endemic 32/252 (12.7)	4.33	1.74–10.77	0.002 (0.012)	Dominant and additive
		Localized lesion 3/25 (12)	Non-endemic 32/252 (12.7)	5.50	1.84–16.45	0.002 (0.016)	Dominant
		Localized lesion 3/25 (12)	Non-endemic 32/252 (12.7)	5.32	1.85–15.24	0.002 (0.021)	Additive
^*^3A2A	*GCRTTTKCG*	Patient 25/292 (8.6)	Non-endemic 43/252 (12.8)	0.46	0.25–0.85	0.014 (0.033)	Dominant
		Patient 25/292 (8.6)	Non-endemic 43/252 (12.8)	0.49	0.27–0.89	0.020 (0.037)	Additive

*Haplotypes represented nine investigated CR1 SNPs (rs6656401, rs3849266, rs2274567, rs3737002, rs11118131, rs11118167, rs17047660, rs4844610, and rs12034383). One-letter, underlined amino acid symbols are given instead of nucleotide substitutions, where appropriate. n, number of chromosomes; OR, odds ratio, corrected for age, ancestry group, and sex distribution; CI, confidence interval*.

* *Fisher's exact test. Dominant model: individuals with the haplotype were more frequent in one of the groups, regardless of whether homozygous or heterozygous. Additive model: homozygote individuals were overrepresented in one of the groups, followed by heterozygotes, meaning that the haplotype presents an additive effect for increasing resistance/protection against the disease*.

### sCR1 Levels and Susceptibility to Fogo Selvagem

**Figure 3:**
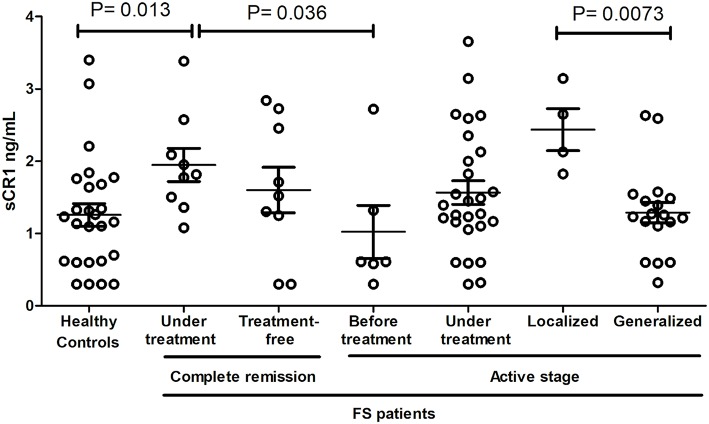
sCR1 serum levels in endemic controls and FS patients. *P*-values were obtained with the Mann-Whitney test. Interquartile median and range are indicated. We excluded two outliers with very high levels (one with 7.14 ng/mL in controls and another with 10.2 ng/mL in FS patients with active disease, but under treatment).

sCR1 levels differed according to treatment and disease manifestations but not according to the genotypes of the investigated SNPs and haplotypes (Figure 3). Patients in complete remission on treatment had higher sCR1 levels (median 1.82 ng/mL) than did controls and patients that had not yet initiated therapy (medians 1.23 and 0.61 ng/mL, *P* = 0.013 and 0.036, respectively). On the other hand, there was no difference between sCR1 levels in controls and patients with active fogo selvagem on treatment (medians 1.2 ng/mL vs. 1.39 ng/mL, respectively, *P* = 0.188). However, those with localized lesions revealed higher sCR1 levels than did patients with generalized lesions (medians 2.39 ng/mL vs. 1.24 ng/mL, respectively, *P* = 0.0073). The sCR1 levels of patients with localized lesions on immunosuppressive treatment were also significantly higher than in controls (*P* = 0.0112). Of note, no difference in sCR1 levels was found between patients treated with low and high corticosteroid doses (*P* = 0.1897, data not shown).

### CR1 mRNA Expression Levels

**Figure 4:**
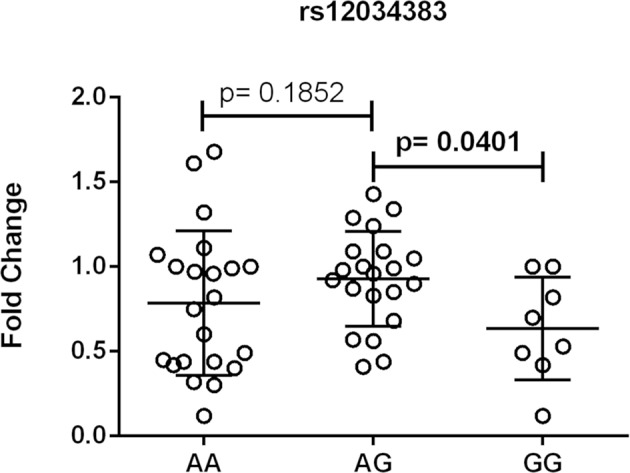
Association between the expression of *CR1* mRNA and *CR1* rs12034383 polymorphism. Fold-change values were calculated through the 2–ΔΔCt method. The horizontal bars in the clusters indicate the median. *P*-values indicate a statistical significance at the 0.05 level and were calculated by Mann–Whitney's test. Scatter plots were constructed from the raw non-normalized, fold change data using GraphPad 6.0 software (La Jolla, California, USA).

**Figure 5:**
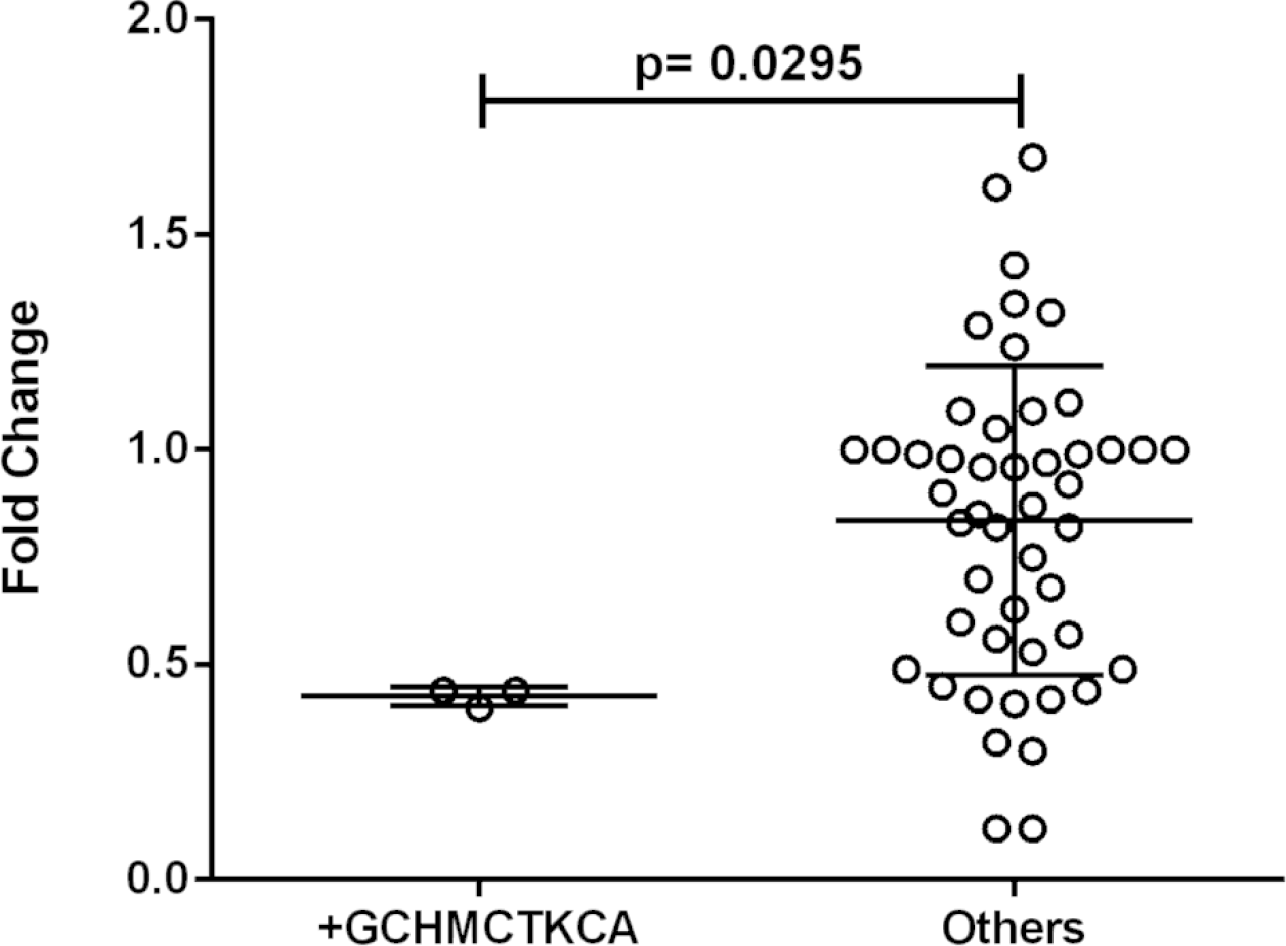
Association between the expression of CR1 mRNA and the ^*^1.3B2B– *GCHMCTKCA* haplotype. +*GCHMCTKCA*: homozygote or heterozygote individuals. Fold-change values were calculated through the 2–ΔΔCt method. The horizontal bars in the clusters indicate the median. *P*-values indicate a statistical significance at the 0.05 level and were calculated by Mann–Whitney's test. Scatter plots were constructed from the raw non-normalized, fold change data using GraphPad 6.0 software (La Jolla, California, USA).

One out of eleven *CR1* SNPs analyzed (rs12034383) was associated with differential mRNA expression levels. Controls with the rs12034383^*^A/G genotype had higher mRNA levels than controls with the rs12034383^*^G/G genotype (median fold changes 0.96 vs. 0.62, respectively, *P* = 0.04) (Figure 4). Carriers of the protective *GTHMCTKCA* haplotype, which includes the rs12034383^*^A allele, presented instead lower mRNA expression (*P* = 0.03) (Figure 5).

## Discussion

CR1 plays a major role in inhibiting the complement system, removing immune complexes, and activating B cells, important events that are deregulated in autoimmune diseases (35, 55, 56). Polymorphisms that alter *CR1* gene expression or protein activity are also common worldwide (57–60) and have been associated with different infectious, autoimmune, and neurological/neurodegenerative diseases (61–67). Those in exon 29 encode antigens of the Knops blood group system, the 22nd system recognized by the International Society of Blood Transfusion (68): e.g., rs3737002 (p.Thr1408Met) defines the York blood antigen and rs17047660 (p.Lys1590Glu), the McCoy blood antigen (69, 70). These polymorphisms may modulate the success of *Plasmodium falciparum* and *Leishmania major*, as well as mycobacteria that invade erythrocytes and macrophages, respectively (71–75). Most of the time, however, disease associations are related to complement subversion/deregulation and consequent alterations in phagocytosis and cytokine release, even in infectious diseases (29, 76–78). For example, in HIV infection, CR1 molecules expressed on erythrocytes bind C3b-opsonized HIV particles. They act as cofactors for factor I conversion of C3b to inactivated iC3b. This allows the virus to survive anti-retroviral therapy and spread as iC3b-opsonized particles, captured by CR2 on B cells, and presented to other lymphocytes (79). In systemic lupus erythematosus, a low CR1 density on neutrophils and other phagocytes causes excessive C3 activation, the release of proinflammatory cytokines, and immune-complex overload (80–82). The risk for Alzheimer's disease increases with the preferential, low expression of a long CR1 isoform, which is ineffective in the removal of beta-amyloid plaques (83–86). Furthermore, *CR1* polymorphisms also modulate the response to eculizumab therapy, which targets complement protein C5 (87).

**Figure 6:**
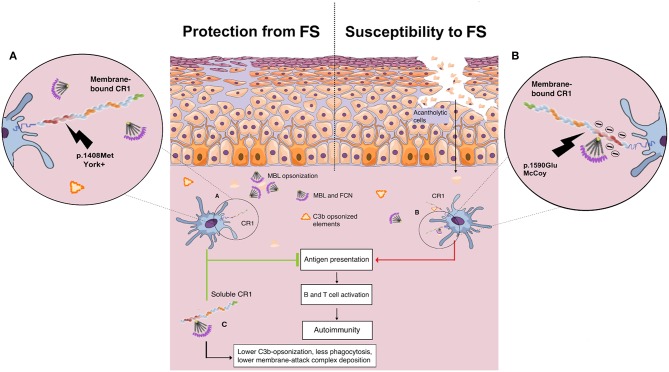
Proposed role of *CR1* polymorphisms of the Knop blood group and soluble CR1 in susceptibility to endemic pemphigus foliaceus. **(A)** The p.1408Met amino acid substitution (York antigen) in the SCR22 module of the protein is proposed to reduce the affinity of CR1 molecules for C1q/collectin/ficolin to IgG1-bound C1q as well to exposed glycosilated/acetylated residues on desmosomes and epithelial cells, decreasing internalization of neoantigens by antigen-presenting cells. **(B)** In contrast, CR1 molecules with p.1590Glu (McCoy antigen) are proposed to have enhanced affinity for C1q/collectin/ficolins due to a negative charge already provided by Glu at the 1595 and 1597 positions, increasing the internalization of cell debris and other opsonized complexes, fostering the autoimmune reaction by renewing the presentation of self-peptides, and consequent B and T cell activation. **(C)** The soluble CR1 molecule has anti-inflammatory properties. As cell membrane-bound CR1, sCR1 acts as a cofactor for the Factor I-mediated cleavage of soluble/bound C3b and C4b, reducing opsonization and phagocytosis. Furthermore, sCR1 and exosomal-bound CR1 are unable to mediate the LHR-D-mediated internalization of opsonized complexes by phagocytes, inhibiting both complement activation, and complement-driven phagocytosis (by the author).

Here, the York blood group, encoded by the CR1^*^3B2B haplotype, was associated with higher protection against FS. The p.1408Met amino acid substitution (rs3737002^*^T) is responsible for this antigen and is considered as “probably damaging” (a Polyphen-2 score of 0.9 or higher and a CADD score of 19.52). This substitution modifies the first SCR in the LHR-D module (SCR 22), responsible for the recognition of the initiation molecules of the classical (C1q) and lectin (MBL and FCNs) pathways, and may reduce the recognition of IgG1-bound C1q as well as of MBL/FCNs (32) bound to exposed glycosilated/acetylated residues on desmosomes and epithelial cells, decreasing internalization of neoantigens by antigen-presenting cells (Figure 6).

As expected, the most ancestral ^*^1 haplotype, encoding p.1408Thr, seems to increase susceptibility to FS. Interestingly, although this association was found by comparing patients with non-endemic controls, a *CR1* haplotype with p.1408Thr was found to increase FS susceptibility 1.4x within the endemic population in our previous study (37). Furthermore, the same haplotype has recently been associated with increased susceptibility to the more severe multibacillary clinical forms of leprosy (25).

On the other hand, the p.1408Met amino acid substitution was also recently associated with susceptibility to idiopathic pulmonary fibrosis (88). Affected patients present an increase of serum proteins of the complement cascade and higher complement activation in the lung (89). The same allele was also reported to increase the susceptibility to late-onset Alzheimer's disease in Han Chinese (90). Compared to the York antigen, the opposite is expected to occur with the amino acid substitution that creates the McCoy “b” antigen in SCR25, namely p.Lys1590Glu (rs17047660^*^G). This SNP is also deemed to be damaging (although with a low Polyphen score: 0.597) or deleterious, according to SIFT (score of 0.02). It increases the amount of glutamic acid residues in a sequence of only 10 residues, strengthening the negative charge already provided by Glu at the 1595 and 1597 positions and thus most probably increasing the affinity of this LHR-D domain for C1q/MBL/FCNs (91). This could explain the association found with increased susceptibility to FS, since it could potentially increase the capture of complement-opsonized elements, their internalization, and consequent B cell activation (Figure 6). The association found with the McCoy antigen (p.1590Glu), however, did not resist correction for demographic factors, which may stem from its higher frequency in African-derived populations and the small sample size of this study. Interestingly, homozygosity for p.1590Glu was also associated with higher susceptibility to repetitive malaria-associated seizures in patients with cerebral malaria (66). The susceptibility associations found for the intronic polymorphisms rs6656401^*^G and rs3849266^*^T are in accordance with our previous results (37), but the latter may also result from strong linkage disequilibrium with rs3737002.

Last, but not least, we found a protective association with the ^*^3A2A haplotype, containing the p.1208Arg (rs2274567^*^G) amino acid substitution. Among the investigated SNPs, this allele was, as expected, also associated with FS resistance. However, both associations only occurred when comparing patients with non-endemic controls. Thus, we recommend caution in interpreting these results until they are replicated in an independent sample.

In accordance with the proposed anti-inflammatory role of the York antigen in disease resistance, we found an association of higher levels of sCR1, a molecule known for its anti-inflammatory properties (92), with less severe clinical presentation of FS, disease remission, and corticoid therapy. This leads us to suggest that sCR1 plays a protective role in the evolution of the disease. The soluble CR1 molecule shares all LHR domains with exosomal-bound CR1 and cell membrane-bound CR1, as well as all functions mediated by LHRs A-C, including the ability to cleave C4b, accelerating the decay of C3 and C5 convertases, and to cleave C3b, reducing opsonization and phagocytosis (27, 93). Nevertheless, sCR1 and exosomal-bound CR1 are unable to mediate the LHR-D-mediated internalization of opsonized complexes by phagocytes, thus acting as a “sink” for initiation molecules of the complement cascade and inhibiting both complement activation and complement-driven phagocytosis (92). Taking into account that human sCR1 has been demonstrated to reduce acute inflammation and autoimmunity, even preventing disease progression in a rat arthritis model (94, 95), and as the potential application of sCR1 in therapeutic settings has been shown (96), its role in susceptibility to pemphigus and its clinical evolution might be highly relevant. A longitudinal follow-up study would be necessary to find out whether patients with localized lesions that have progressed to the generalized form also present a decline in sCR1 concentration. In the present study, *CR1* genotypes were not correlated with sCR1 serum levels, which might rely on the fact that the sCR1 form does not result from alternative splicing of the gene but from proteolytic cleavage (31).

*CR1 g*ene expression was higher in healthy individuals with the genotype *A/G* (rs12034383) than in *G/G* homozygotes. This latter genotype is also associated with impaired removal of amyloid Aß in the cerebrospinal fluid of Alzheimer patients compared with *A/A* homozygotes (83). Interestingly, the recombinant ^*^1.3B2B (*GCHMCTKCA*) haplotype, containing the proposed protective York antigen (p.1408Met), was associated with lower gene expression, lending support to the hypothesis that low CR1 levels would protect against the production of autoantibodies, as previously discussed.

In conclusion, our data lead us to suggest that *CR1* polymorphisms of the Knops blood group modulate susceptibility to FS. Furthermore, higher sCR1 levels may limit FS lesions and promote/accelerate disease remission. CR1 may thus be regarded as a candidate for new therapeutic interventions in the disease.

## Data Availability Statement

The raw data supporting the conclusions of this manuscript will be made available by the authors, without undue reservation, to any qualified researcher.

## Ethics Statement

The studies involving human participants were reviewed and approved by The National Committee for Ethics in Research (CONEP 02727412.4.0000.0096, protocol 505.988). Written informed consent to participate in this study was provided by the participants' legal guardian/next of kin.

## Author Contributions

AB administered the project and supervised this work. AB and LO contributed to the conception of the work and curated and analyzed the data. LO, GK, AS, RN, TF, AF, and MW performed the investigation. CC obtained and prepared samples. LO and GK further provided methodological input by developing the multiplex PCR-SSP method used in this study. MP-E provided the samples, and ES and HB provided resources for analysis. AB, MP-E, ES, and HB acquired the funding. LO drafted the manuscript, which was further edited by AB. All authors revised the work critically for intellectual content and approved the final version of the work.

### Conflict of Interest

The authors declare that the research was conducted in the absence of any commercial or financial relationships that could be construed as a potential conflict of interest.

## Supplementary Material

Supplementary Figure 1 Pairwise linkage disequilibrium plot based on investigated *CR1* SNPs. Colors are indicative of D'/logarithm of odds (LOD), and values correspond to r^2^. Bright red represents LOD score for LD ≥ 2 and D = 1, shades of pink/red represent LOD ≥ 2 and D < 1, blue represents D = 1 but LOD < 2, and white squares represent LOD < 2 and D < 1. **(A)** Euro-Brazilian patients. **(B)** Euro-Brazilian controls. **(C)** Afro-Brazilian patients. **(D)** Afro-Brazilian controls. The SNPs rs12034598 and rs1746659 are not represented in the plot because they were not genotyped for all individuals.

Supplementary Table 1 PCR conditions. *HGH* was used as an internal amplification control for simple PCR 1, 2 and multiplex 1; *HLA-E* was used as an internal amplification control for multiplex PCR 2; Simple PCR 1: *CR1* forward primer-1 pair detects rs6656401 alleles; Simple PCR 2: *CR1* forward primer-1 pair detects rs12034598 alleles and *CR1* reverse primer-1 pair detects rs1746659 alleles; Multiplex PCR 1: *CR1* forward primer-1 pair detects rs3849266 alleles and *CR1* reverse primer-1 pair detects rs2274567 alleles; and *CR1* forward primer-2 pair detects rs4844610 alleles and *CR1* reverse primer-2 pair detects rs12034383 alleles; Multiplex PCR 2: CR1 forward primer-1 pair detects rs3737002 alleles and *CR1* reverse primer-1 pair detects rs11118131 alleles; and *CR1* forward primer-2 pair detects rs11118167 alleles and *CR1* reverse primer-2 pair detects rs17047660 alleles; C, Celsius; min: minute; sec: seconds.

Supplementary Table 2 Allele and genotype distributions of *CR1* polymorphisms investigated in this study.
