# Supplementary figures and images for: Complement Receptor 1 (CR1, CD35) Polymorphisms and Soluble CR1: A Proposed Anti-inflammatory Role to Quench the Fire of “Fogo Selvagem” Pemphigus Foliaceus
Source: Front Immunol. 2019 Nov 22;10:2585. doi: 10.3389/fimmu.2019.02585 (PMC6883348; doi:10.3389/fimmu.2019.02585)

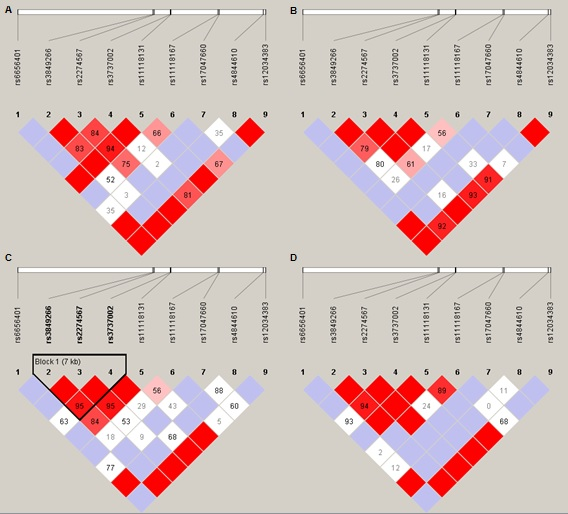

Supplement: Supplementary Figure 1 — Pairwise linkage disequilibrium plot based on investigated CR1 SNPs. Colors are indicative of D'/logarithm of odds (LOD), and values correspond to r2. Bright red represents LOD score for LD ≥ 2 and D = 1, shades of pink/red represent LOD ≥ 2 and D < 1, blue represents D = 1 but LOD < 2, and white squares represent LOD < 2 and D < 1. (A) Euro-Brazilian patients. (B) Euro-Brazilian controls. (C) Afro-Brazilian patients. (D) Afro-Brazilian controls. The SNPs rs12034598 and rs1746659 are not represented in the plot because they were not genotyped for all individuals. [file Image_1.tiff]
